# An inexpensive and rapid method for removal of multiple stripped locking screws following locking plating: A case report

**DOI:** 10.1016/j.ijscr.2019.03.046

**Published:** 2019-03-30

**Authors:** Won Ro Park, Jae Hoon Jang

**Affiliations:** a Department of Orthopaedic Surgery, Dong-Eui Medical Center, Busan, Republic of Korea; b Department of Orthopaedic Surgery, Trauma Center, Bio-Medical Research Institute, Pusan National University Hospital, Busan, Republic of Korea

**Keywords:** Locking plate, Stripped locking screw, Locking screw removal

## Abstract

**Introduction:**

Removal of stripped or jammed locking screws involves several difficulties. Especially when two or more screws are involved, their removal is a more challenging and time-consuming process because the various reported techniques have certain limitations.

**Case presentation:**

Herein, we report a case in which five stripped screws were removed using a novel technique.

**Discussion:**

Many techniques to remove damaged locking head screw were introduced, however in case with multiple screws were involved, they are limited to use. In the case, screw extractor, although it is not a medical device, is helpful in terms of time and cost.

**Conclusion:**

In case with the failure of removal of multiple locking screws, our technique using non-medical screw extractor can be a good option.

## Highlights

• Removal of stripped or jammed locking screws involves several difficulties.
• Especially when two or more screws are involved, the various reported techniques have certain limitations.
• When failed to remove multiple locking screws, using non-medical screw extractor can be an inexpensive, rapid and effective option.

## 1 Introduction

Locking plates are widely used owing to their higher fixation strength than conventional plates, but removal of locking screws is challenging when the screw head is damaged [[1], [2], [3], [4]]. In this situation, various techniques can be used such as use of special instruments (e.g., conical extraction screws), plate cutting [2] or removal of the screw head using a burr [3]. On the other hand, if only a single screw head is damaged, the screw can be removed by simply rotating the plate [5]. However, all these methods require additional time and cost, and these methods have limited use when multiple screws are damaged. Similarly, we encountered some difficulties during the removal of a broken locking screw head. Therefore, the aim of this study was to propose an easier and less expensive technique for the removal of multiple damaged locking screw heads and to present a literature review.

This case report has been reported in line with the SCARE criteria [6].

## 2 Case presentation

**Fig. 1:**
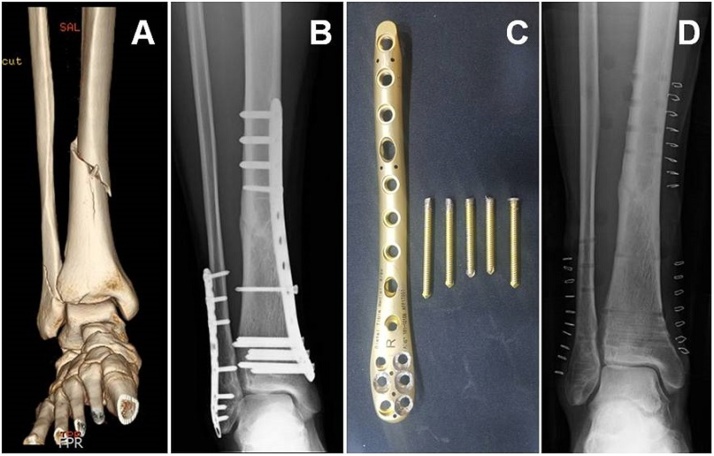
(A) 29-year-old male sustained fractures of the right tibial shaft and lateral malleolus, and underwent surgical treatment using a locking plate at another hospital. (B) Then, he was transferred to our hospital, and following identification of bony union, plate removal was scheduled. (C) Intraoperatively, multiple heads of the locking screw were damaged and could not be removed. Thus, we used a screw extractor (IRWIN^®^, Huntersville, NC, USA), a non-medical instrument that had been sterilized. Through drilling, the plate was separated from the screws and could be removed first. The remaining shafts of the screws were removed with a vice grip. (D) Postoperative radiograph reveals that the plate and screws were removed without substantial complications.

A 29-year-old male with a history of right tibial shaft and lateral malleolus fractures following a fall injury underwent open reduction and internal fixation with a locking plate for the fibula fracture and minimally invasive percutaneous osteosynthesis for the tibial shaft fracture at another hospital. Postoperatively, the patient was followed up at our hospital for 1 year due to residential issues, and the removal of the plate was scheduled after confirmation of bone union. According to the previous hospital’s medical records, a 9-hole locking plate (APIS^®^, Gwangju, Republic of Korea) was used for the distal tibia, with three 5.0-mm locking screws for proximal fixation and six 3.5-mm locking screws for distal fixation. Under spinal anesthesia, a plate removal surgery was performed, following which the fibular plate was easily removed. However, five of the six distal locking screws were damaged, and they could not be removed with a screwdriver. This situation was explained to the patient during the operation, and we could confirm that the patient wanted to remove the plate irrespective of the amount of time required. At the time, a tourniquet was used for approximately 1 h, and screw removal was attempted using a conical extraction screw, but only one of the six screws could be removed. Previously proposed techniques requiring additional skin incisions were not considered because there were multiple damaged screws that could not be removed, these techniques would require extended amount of time for removal, and the surrounding soft tissues could be damaged. Hence, we decided to use a screw extractor (IRWIN^®^, Huntersville, NC, USA), a non-medical instrument, following sterilization with ethylene oxide. In brief, 6-mm drill bit of the extractor was used, four screw heads were drilled, the locking screws and locking plate were separated, and the plate was removed. Of the remaining four screws, one was removed using a vice grip; the remaining three screws could not be removed with the vice grip owing to a short extruded portion. A hollow reamer could not be used because the three screws were adjacent to each other and the reamer could cause a large bone defect; hence, we created sufficient space around the screws by forming small holes around them with a 1.8-mm K-wire, following which all screws could be removed using the vice grip. After saline irrigation, debridement of the soft tissues contaminated with metal debris was performed. The sizes of the metal debris were found to be relatively large, which facilitated easier debris removal. With 2-h use of a tourniquet, all implants could be removed and the skin could be closed (Fig. 1). Postoperatively, no complications were noted during physical and radiological examinations. Remarkably, no problems were noted at the final outpatient visit at 3 months after discharge; therefore, follow-up was terminated. The patient had not visited an outpatient clinic for more than a year since the last follow-up, thus we thought that he had no specific complications including infection and re-fracture.

## 3 Discussion

Locking plates are widely used as they present various advantages including high fixation strength. As a result, the number of surgeries for locking plate removal has been increasing, and the frequency of locking head screw damage is expected to increase. During metal plate removal, most head screw heads are damaged with 3.5- and 5.0-mm locking screws, with incidence rates of 5.7%–10.3% and 0%–0.7%, respectively [5,7]. We believe such damages usually occur with 3.5-mm locking screws because the hexagonal recesses of these screws are more vulnerable by overtorque. Moreover, in the present case, 5.0-mm proximal screws were removed without any complications, whereas the distal 3.5-mm screws were damaged. The failure of screw removal can be due to several causes such as incorrect insertion angle during the first operation; avoiding the use of a torque limiting screwdriver during the final step of screw insertion, resulting in excessive tightening between the metal plate and screw head (jamming); and use of a worn-out screwdriver for screw insertion. In surgeries involving the use of a locking plate, it is important to check whether the locking sleeve is inserted in the correct position on the metal plate and the direction should be checked with an image intensifier when the direction of screw insertion is uncertain. In addition, during the final tightening of the locking screw, it is important to use a torque limiting wrench to prevent excessive tightening with excessive strength. If the hexagonal recess is damaged during tightening, it should ideally be documented in the operation records to preoperatively prepare for this situation, even if the removal surgery is performed by another surgeon. During removal surgeries, the wear state of the driver tip should be repeatedly checked, and a new driver should be arranged to prepare for damages of locking screws.

**Fig. 2:**
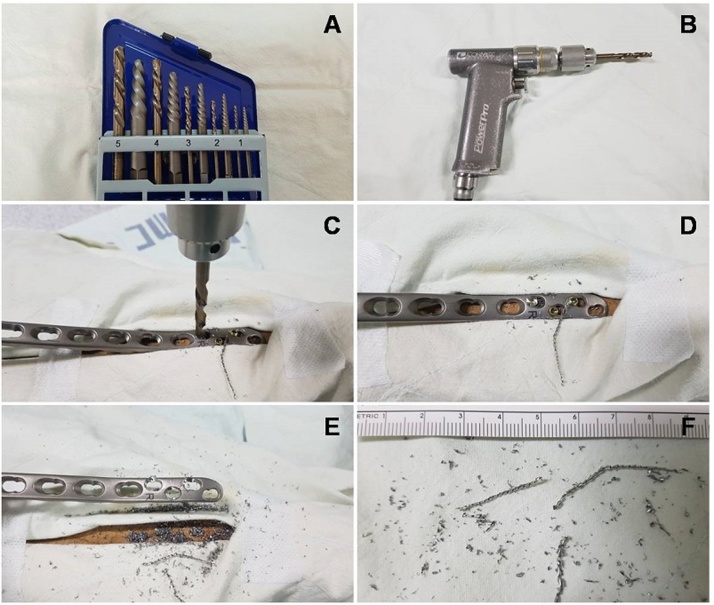
We measured the time required to remove the screws and the size of metal debris through our technique. (A) The drill bits in a screw extractor set (IRWIN^®^, Huntersville, NC, USA) are made of cobalt and not medical instruments. (B) A 6-mm diameter drill bit for the removal of 3.5 mm locking screws was held to a Jacobs Chuck of pneumatic-powered tool. (C) A plate was fixed in a wooden bar with the three locking screws whose heads were deliberately damaged. Further, we drilled the heads in a counterclockwise manner. (D) One head of the locking screw was then removed, which required less than a minute. (E) Next, 2 more heads removed, which required approximately 2 min to the separation of the plate and screws. (F) Upon observation of metal debris, the size of particles was found to be relatively large to remove.

Screw damages can occur even when considerable attention is paid; hence, various special instruments and techniques have been introduced for the removal of damaged locking screw heads. Use of conical extraction screws can be considered, but the success rate of this instrument is less than 50% [2,8] and only a single use is recommended; hence, the instrument is considered relatively expensive, making it difficult for small and medium-sized hospitals to purchase and use this instrument. Further, cutting a part of the metal plate or removing the screw head requires instruments such as cutting discs and burrs, resulting in complications such as a possible heat injury or iatrogenic injury of surrounding tissues, generation of difficulty in metal debris removal, and requirement of an additional incision [2,3,9]. In addition, these instruments are not reusable and are expensive. Plate bending and rotation require an additional incision, and when a metal plate is thick or multiple screws are damaged, this method is not considered feasible [7]. Multiple hole construction around a metal plate and pushing of the plate are not possible when multiple screws are damaged and when the plate is not a combi-hole plate [10]. Further, metal plate removal by pulling the plate upward following weakening of the hexagonal recess with a drill cannot be used in the case of multiple screw damages, and during the removal of the plate, there is a possibility of a fracture [5]. As such, when multiple screws are damaged, previously reported techniques present limitations and require tremendous effort and time. The technique used by us for the removal of multiple screws has certain advantages, including a relatively cheap price (the price of this product currently is 34.95 US dollars on eBay), reusability, less time requirement, no necessity of additional incisions, and generation of relatively large metal debris, reducing soft tissue contamination (Fig. 2). Use of non-medical instruments includes a difficult, but the Ministry of Public health and Welfare of Korea stated that in cases wherein use of non-medical devices is inevitable during metal plate removal, their use is not considered a violation of medical law.

Keen attention during the initial operation is vital, but preparation of special instruments for use in cases of screw removal failure and knowledge of different removal techniques are deemed necessary. As in this case report, when other techniques cannot be used owing to multiple locking screw head damages, the method proposed in this study can prove to be valuable.

## 4 Conclusion

In case with the failure of removal of multiple locking screws, our technique using non-medical screw extractor (6-mm drill bit) following sterilization can be inexpensive and rapid option to remove them.

## Conflicts of interest

The authors declare no conflict of interest.

## Sources of funding

This work was supported by a clinical research grant from Pusan National University Hospital in 2018.

## Ethical approval

No Institutional Review Board is required for publication of a case report in our institution.

## Consent

Informed consent was obtained from the patient for publication of this case report and accompanying figures.

## Author contribution

Won Ro Park – performing the surgery, study concept, data collection, advised and designed the study.

Jae Hoon Jang – writing the paper.

## Registration of research studies

N/A.

## Guarantor

Jae Hoon Jang.

## Provenance and peer review

No commissioned, externally peer-reviewed.
